# Skeletal stem/progenitor cells provide the niche for extramedullary hematopoiesis in spleen

**DOI:** 10.3389/fphys.2023.1148414

**Published:** 2023-03-17

**Authors:** Helen C. O’Neill, Hong Kiat Lim

**Affiliations:** Clem Jones Centre for Regenerative Medicine, Bond University, Gold Coast, QLD, Australia

**Keywords:** skeletal stem/progenitor cells, stromal cells, extramedullary hematopoiesis, hematopoietic niche, spleen

## Abstract

In bone marrow, the niche which supports hematopoiesis and nurtures hematopoietic stem cells (HSCs) contains perivascular reticular cells representing a subset of skeletal stem/progenitor cells (SSPCs). These stromal cells which provide the niche are lost or become inadequate during stress, disease or ageing, such that HSCs leave bone marrow and enter spleen and other peripheral sites to initiate extramedullary hematopoiesis and particularly myelopoiesis. Spleen also maintains niches for HSCs under steady-state conditions, evident since neonatal and adult spleen contain HSCs in low number and provide low-level hematopoiesis. In spleen, HSCs are found in the sinusoidal-rich red pulp region also in the vicinity of perivascular reticular cells. These cells resemble to some extent the known stromal elements reflecting HSC niches in bone marrow, and are investigated here for their characteristics as a subset of SSPCs. The isolation of spleen stromal subsets and the generation of cell lines which support HSCs and myelopoiesis *in vitro* has led to the identification of perivascular reticular cells which are unique to spleen. Analysis of gene and marker expression, as well as differentiative potential, identifies an osteoprogenitor cell type, reflective of one of several subsets of SSPCs described previously in bone, bone marrow and adipose tissue. The combined information supports a model for HSC niches in spleen involving perivascular reticular cells as SSPCs having osteogenic, stroma-forming capacity. These associate with sinusoids in red pulp to form niches for HSCs and to support the differentiation of hematopoietic progenitors during extramedullary hematopoiesis.

## Introduction

Mesenchymal or skeletal stem and progenitor cells have now been described in many tissue sites including bone and bone marrow (Chan et al., 2009; Lv et al., 2014), and also within the vasculature of many tissues including kidney, heart and adipose tissue (Craig et al., 2022). The definitive skeletal stem cell was recently isolated from growth plates of adult and fetal bone of humans and mice, and has osteogenic, chondrogenic and stroma forming capacity (Chan et al., 2015; Chan et al., 2018). The classification of skeletal stem/progenitor cells (SSPCs) now replaces the spurious ‘mesenchymal stem cells’ commonly isolated as fibroblast-like cells which grow out of cultures of bone marrow (Pittenger et al., 1999; Pittenger et al., 2019). While early studies showed that culture-derived mesenchymal precursors could form stroma which support hematopoietic stem cell (HSC) maintenance (Calvi et al., 2003; Zhang et al., 2003), more recent studies confirm an important role for these cells in the formation and regulation of HSC niches and hematopoiesis in bone marrow (Comazzetto et al., 2021). Distinct subsets of perivascular reticular cells in bone marrow have now been described as important stromal elements of the HSC niche which supports hematopoiesis (Sacchetti et al., 2007; Corselli et al., 2013; Crane et al., 2017). More recent identification of subsets of SSPCs now confirms a heterogeneity of subsets isolatable from either the skeletal growth plate (Chan et al., 2015; Chan et al., 2018), or as bone marrow subsets of skeletal progenitors (Zhou et al., 2014; Baccin et al., 2020; Tournaire et al., 2020; Matsushita et al., 2021; Shen et al., 2021), and also within the pericyte and adventitial cell populations that surround blood vessels in vascularised tissues (Sacchetti et al., 2016; Wang et al., 2020; Xu et al., 2021; Craig et al., 2022). It is therefore of interest to determine whether the same SSPCs reside in bone and bone marrow as in other tissue sites, and whether their role in supporting hematopoiesis is restricted to bone marrow or is a feature of many tissue sites. A recent study has identified distinct bone-forming capacity of SSPCs located in bone marrow as opposed to periosteum (Jeffery et al., 2022). Periosteal SSPCs contribute to only transient formation of trabecular bone at fracture sites, but regenerate stromal cells expressing hematopoietic niche factors (Jeffery et al., 2022). Their important role in HSC niche formation, suggests a tight linkage between the mesenchymal and hematopoietic systems at the level of stem cell maintenance.

While bone marrow is the main site for hematopoiesis in adults, increasing evidence points to a role for spleen in the maintenance and differentiation of HSCs. Analysis of HSC niches in spleen and delineation of the mechanisms by which they regulate hematopoiesis, is important in terms of utilisation of spleen as an alternate site for hematopoiesis when bone marrow is compromised by disease or ageing. A history of work from this lab has considered the potential for re-engineering HSC niches in spleen in order to increase hematopoietic cell production (Tan and Watanabe, 2017; O'Neill et al., 2019). If unique stromal cells can be isolated and used to expand HSCs *in vitro*, or provided as an ectopic niche for the same purpose *in vivo*, then the potential exists to enhance hematopoiesis. This article investigates the stromal cells which support hematopoiesis in spleen, and the evidence that perivascular reticular cells which provide the niche for HSCs are reflective of a subset of mesenchymal SSPCs. Regeneration or expansion of HSC niches could represent future therapy for patients undergoing HSC transplantation, myeloablative treatment or involution of lymphoid tissue with ageing. It is therefore important to fully characterise SSPCs in spleen, their growth and differentiative capacity, and the mechanisms by which they support the maintenance of HSCs.

### The hematopoietic stem cell niche

Schofield introduced the concept of the hematopoietic ‘niche’ in the 1970s after observing that once HSCs were removed from the bone marrow microenvironment they quickly lost capacity to self-renew and to reconstitute the hematopoietic system (Schofield, 1978). The ‘niche’ is now described as a microenvironment comprising non-hematopoietic stromal cells, extracellular matrix and soluble regulatory factors that contribute to stem cell dormancy, quiescence, self-renewal and differentiation, so regulating the fate of HSCs (Crane et al., 2017; Comazzetto et al., 2021; Sánchez-Lanzas et al., 2022). Over time, three main stromal cell types were found to contribute to the HSC niche in bone marrow, namely, endosteal, vascular and perivascular cells (Kiel and Morrison, 2008; Bianco, 2011; Nagasawa et al., 2011; Corselli et al., 2013). It is now clear that interconnected cellular microenvironments provide the niche for HSCs in adult tissue. HSC in bone marrow have been associated most commonly with sinusoidal blood vessels, less commonly with arterioles, with only small numbers of primitive HSCs associated with the endosteum of bone (Crane et al., 2017). HSCs in the vicinity of the vasculature associate with reticular stromal cells which provide CXCL12 for HSC maintenance (Sugiyama et al., 2006a; Ding et al., 2012; Greenbaum et al., 2013). A dichotomy of periarteriolar and perisinusoidal reticular cells (Kunisaki et al., 2013; Acar et al., 2015) along with endothelial cells provide a source of stem cell factor (SCF) for HSC proliferation (Ding et al., 2012; Greenbaum et al., 2013). Current data no longer supports a periarteriolar niche for quiescent HSC (Kokkaliaris et al., 2020), although HSC dependent on periarteriolar niches have been reported during postnatal development (Isern et al., 2014; Asada et al., 2017a). Recent modelling identifies the motility of HSC within the bone marrow niche (Upadhaya et al., 2020), and the close proximity of HSC to a multitude of cell types (Gomariz et al., 2018).

A role for osteoblastic cells in HSC maintenance in bone marrow was first demonstrated in studies varying the number of these cells experimentally (Calvi et al., 2003). Constitutive expression of an active form of parathyroid hormone (PTH) or the PTH-related protein receptor (PPR) gave a marked increase in both the number of osteoblastic cells and the number of HSCs (Calvi et al., 2003). Osteoblasts maintain HSCs through secretion of cytokines like angiopoietin-1 (ANGPT1), thrombopoietin (THPO) and osteopontin (SPP1) which bind to cell surface receptors on HSCs (Nilsson et al., 2005; Qian et al., 2007; Lilly et al., 2011). They also express Jagged 1 which engages with Notch receptors on HSCs, so inhibiting differentiation and enhancing HSC self-renewal (Calvi et al., 2003; Qian et al., 2007; Lilly et al., 2011). Similarly, *Spp1* ^−/−^ mice showed a marked increase in the number of HSCs cycling, consistent with osteopontin (SPP1) as an inhibitor of HSC proliferation (Nilsson et al., 2005). However, the direct involvement of osteoblastic cells was challenged when researchers failed to observe a change in HSC numbers after depletion of osteoblasts using ganciclovir treatment or biglycan deficiency (Visnjic et al., 2004; Kiel et al., 2007). *In vivo* imaging studies also revealed few HSCs in direct contact with bone cells (Lo Celso et al., 2009). A vascular niche was also described in the vicinity of blood vessels in bone marrow. This is associated with rapid mobilisation of HSCs into the bloodstream after administration of granulocyte colony stimulating factor (G-CSF) as a mobilising agent (Kiel et al., 2005). Vascular niches also function to support hematopoiesis during embryogenesis since HSCs self-renew and differentiate at a stage of foetal development when bone marrow cavities are not yet formed (Huber et al., 2004). The role of vascular endothelial cells as regulators of hematopoietic integrity was demonstrated by conditionally deleting the signalling molecule vascular endothelial growth factor receptor 2 (VEGFR2) in adult *Vegfr2* ^*−/−*^ mice. This impeded development of sinusoidal endothelial cells after irradiation and prevented reconstitution of the hematopoietic system (Hooper et al., 2009).

Mesenchymal perivascular reticular cells expressing high levels of the chemokine CXCL12 have now been identified as probably the most important element of the HSC niche in bone marrow. Several subsets were first characterised and described variably as CXCL12-abundant reticular (CAR) cells (Sugiyama et al., 2006b), nestin^+^ mesenchymal stem cells (Mendez-Ferrer et al., 2010) and leptin receptor^+^ stromal cells (Ding et al., 2012). CAR cells in bone marrow were characterised as bipotent adipo-osteogenic progenitors, developing around sinusoids and maintaining HSCs in an undifferentiated state (Sugiyama et al., 2006b; Omatsu et al., 2010). Conditional ablation of CAR cells using transgenic mice with the diphtheria toxin receptor gene inserted into *Cxcl12* led to a reduction in both HSCs and myeloid differentiation (Omatsu et al., 2010). HSCs have also been localised to nestin^+^ mesenchymal stem cells situated near arterioles in bone marrow (Mendez-Ferrer et al., 2010). On conditional ablation of these cells from mice, HSC numbers decreased so indicating their importance in forming a perivascular niche in bone marrow (Mendez-Ferrer et al., 2010). Leptin receptor^+^ stromal cells expressing high levels of CXCL12 were identified as perivascular cells surrounding sinusoids. All three described subsets reflect an important source of SCF (Ding et al., 2012), a cytokine that signals the c-Kit tyrosine kinase receptor on hematopoietic stem/progenitor cells (McNiece and Briddell, 1995). Loss of the HSC pool in *Scf* ^*−/−*^ mice, highlights the importance of SCF produced by perivascular reticular cells in HSC maintenance (Ding et al., 2012). It is now however very clear that this population is heterogeneous and that several distinct cell types may exist each with distinct roles in hematopoiesis.

Many studies now support the identification of bone marrow stromal cells as subsets of perisinusoidal and periarteriolar stroma. The former commonly have adipogenic differentiative potential and form perisinusoidal niches for HSC, while periarteriolar stromal cells show osteogenic differentiative potential and increase in number upon mechanical stimulation or fracture of bone (Zhou et al., 2014; Baccin et al., 2020; Shen et al., 2021). Transcriptional profiling of bone marrow stromal cells has revealed considerable remodelling under stress which impacts hematopoietic output (Tikhonova et al., 2019), in particular skewing of cells towards adipogenesis. Recently a cell-based protein expression analysis of stromal cells in homeostatic bone marrow revealed 28 distinct subsets of cells of which 14 expressed regulators of hematopoiesis (Severe). Most subsets were sensitive to irradiation conditioning used for HSC transplantation, except some CD73-expressing stromal cells which express factors which enable HSC engraftment (Severe et al. (2019).

### The spleen in hematopoiesis

A hematopoietic role for spleen was first indicated by early evidence documenting survival of lethally irradiated mice where the spleen had been shielded with lead (Rugh and Grupp, 1960). We now understand that during adult life, spleen undergoes extramedullary hematopoiesis at times of physiological stress or infection (Kim, 2010), or when bone marrow is compromised through disease or damage (Yamamoto et al., 2013). Movement of HSCs and hematopoietic progenitors between bone marrow, blood and spleen occurs with induction of pregnancy (Nakada et al., 2014), such that spleens of pregnant mice contain higher numbers of HSCs and also expanded HSC niches (Nakada et al., 2014; Inra et al., 2015). The peripheral blood of pregnant mice also contains increased numbers of HSCs, multipotential progenitors, and myeloid progenitors (He et al., 2009; Oguro et al., 2017). When G-CSF is used to mobilize HSCs out of bone marrow, into blood and then spleen (Morrison et al., 1997), migrating HSCs localise around the sinusoids in the splenic red pulp region (Kiel et al., 2005). This is also seen with blood loss and pregnancy (Inra et al., 2015).

Extramedullary hematopoiesis also occurs as a natural process during fetal development which is later activated during pregnancy, stress and infection (Kim, 2010). The active nature of the process is evident since the low number of HSCs present in murine spleen in the steady-state increases quickly following inflammation (Wolber et al., 2002; Massberg et al., 2007). Passive hematopoiesis also occurs in steady-state adult spleen or following bone marrow failure with ageing (Kim, 2010), and several species, including pigs, baboons and humans retain a low number of HSCs in spleen under resting or steady-state conditions (Dor et al., 2006; Tan and O'Neill, 2010). Moreover, in cell tracing experiments, spleen cells from resting neonatal and resting adult mice can provide hematopoietic reconstitution of lethally irradiated host mice following adoptive transfer (Tan and O'Neill, 2010). Evidence of a role for spleen in steady-state hematopoiesis raises question about splenic niches for HSCs and whether the same niche elements support the maintenance of HSCs in both the resting and inflammatory states.

The dynamic role of spleen in provisioning extramedullary hematopoiesis during stress relies on the rapid expansion of the stromal cells forming the niche (Kiel et al., 2005; Inra et al., 2015; Oda et al., 2018). HSC in spleen have been located in the red pulp region in the vicinity of sinusoids (Inra et al., 2015). Mesenchymal progenitor-like cells expressing Tlx1, a transcription factor for spleen organogenesis, have been described as essential elements of the HSC niche located in the red pulp region (Dear et al., 1995; Oda et al., 2018). Further studies by Inra et al. (2015) identified stromal cells which produce CXCL12 and SCF upon induction of extramedullary hematopoiesis. Tcf21, a marker unique to splenic stromal cells, was used to identify perisinusoidal reticular cells in red pulp proximal to HSC and producing SCF and CXCL12 (Oda et al., 2018). Conditional deletion of *Scf* from endothelial cells and of *Scf* and *Cxcl12* from *Tcf21*-expressing stroma, reduced extramedullary hematopoiesis in spleen without affecting hematopoiesis in bone marrow. Perisinusoidal reticular cells in spleen resemble bone marrow HSC niche elements through their expression of PDGFRα/β and the production of CXCL12 and SCF (Oda et al., 2018), but remain distinct through expression of *Tlx1* and *Tcf21*.

### Hematopoietic support capacity of splenic stroma cells

A history of work in this lab has identified the capacity of specific splenic stromal cells to support hematopoiesis and particularly myelopoiesis *in vitro* (O'Neill et al., 2014)*.* Despite the limitations of *in vitro* analyses using cell lines, the findings of those studies have been highly reproducible over many years and reinforced by different experimental approaches. Long-term cultures of spleen were first shown to support continuous production of myeloid cells arising from progenitors maintained within culture (Ni and O'Neill, 1997; O'Neill et al., 2004). Cell production in cultures depended on a mesenchymal stromal cell layer which proliferated slowly and could be readily maintained (Despars et al., 2004; Despars and O'Neill, 2006a). In particular, stroma-dependent cultures of 6-day old murine spleen support the maintenance of small hematopoietic progenitors, and the continuous production of a distinct class of large, dendritic-like cells which have antigen presenting capacity (O'Neill et al., 2011; Periasamy et al., 2009; Ni and O'Neill, 1999; Periasamy et al., 2013). An original STX3 stromal line was isolated from one culture which had ceased support of myelopoiesis after multiple passages *in vitro* due to loss of progenitors (Ni and O'Neill, 1998). Interestingly, myelopoiesis was again supported when STX3 stroma was overlaid with lineage-depleted (Lin^−^) cells derived from bone marrow which are highly enriched for HSCs and hematopoietic progenitors (Despars and O'Neill, 2006a). A series of studies on cell production identified production of a majority population of myeloid cells as large MHC-II^-^ dendritic-like cells (Periasamy and O'Neill, 2013; Tan et al., 2011). These cells are highly efficient in endocytosis and cross-presentation of antigen for CD8^+^ T cell activation, but do not activate CD4^+^ T cells as do cells of the common dendritic lineage (Periasamy and O'Neill, 2013; Tan et al., 2011). They represent a population of antigen presenting cells unique to spleen. The highly reproducible nature of cell production in long-term cultures and in co-cultures over splenic stroma, is supported by evidence for an *in vivo* equivalent antigen presenting cell subset in murine and human spleen (Hey and O'Neill, 2016).

In order to better characterise spleen stroma and how it supports myelopoiesis *in vitro*, STX3 was cloned to form multiple cloned cell lines (Despars and O'Neill, 2006a; Despars and O'Neill, 2006b). These included the 5G3 clone, as a supporter of *in vitro* hematopoiesis, and 3B5 as a non-supporter. The 5G3 clone supports production of MHCII^−^ dendritic-like cells in a highly reproducible, contact-dependent manner similar to the parent line (Periasamy et al., 2009; Periasamy and O'Neill, 2013). Since co-cultures maintained long-term myelopoiesis, the possibility that they maintain self-renewing HSCs or hematopoietic progenitor cells, was investigated. Various progenitor subsets from bone marrow and spleen were sorted and tested for capacity to seed 5G3 stroma for myelopoiesis. When the Flt3^−^c-Kit^+^Lin^−^Sca-1^+^ subset of long-term HSC from bone marrow, and the Flt3^+^c-Kit^+^Lin^−^Sca-1^+^ subset of short-term HSC were overlaid on 5G3 stroma, production of MHCII^−^ dendritic-like cells was supported (Petvises and O'Neill, 2014a). However, no production was observed in co-cultures overlaid with myeloid dendritic progenitors (MDPs) or common dendritic progenitors (CDPs). These unique spleen-derived dendritic-like cells must therefore derive from a lineage distinct from that of common dendritic cells, plasmacytoid dendritic cells or monocytes (Petvises and O'Neill, 2014b).

It is now clear that the progenitors which seed 5G3 splenic stroma for *in vitro* myelopoiesis reflect HSCs endogenous to spleen, and that the process of myelopoiesis reflects extramedullary hematopoiesis. During development, HSCs and hematopoietic progenitors first appear in murine spleen at embryonic day 18.5, while progenitors of common DC appear at 4 days after birth (Petvises and O'Neill, 2014c). This raises the possibility that hematopoietic progenitors in spleen are laid down during ontogeny, and that myelopoiesis in steady-state adult spleen can occur as an active process not dependent on inflammatory signalling. Hence, we tested whether *in vitro* hematopoiesis in 5G3 co-cultures was dependent on inflammation by assessing the importance of Toll-like receptor signalling to cell production (Periasamy et al., 2013). Co-cultures established with bone marrow progenitors derived from mutant *MyD88* ^*−/−*^ and *Trif* ^*−/−*^ mice, which lack the adapter proteins MyD88 and TRIF crucial for Toll-like receptor signalling, were found to be equivalent supporters of myelopoiesis with production of MHCII^−^ dendritic-like cells. Myelopoiesis *in vitro* occurs independently of Toll-like receptor signalling and inflammation (Periasamy et al., 2013).

One model is that the splenic stromal microenvironment supports restricted and directed differentiation of endogenous hematopoietic progenitors to give antigen presenting cells unique to the spleen microenvironment. Indeed, studies to date on the *in vivo* tissue distribution of these cells confirms them to be a novel subset limited to spleen (Tan et al., 2011). Indeed, such an MHCII^−^ antigen presenting cell type could be positioned to receive antigen entering spleen from blood for rapid induction of a CD8 T cell response to manage blood-borne infections or cancers. Antigen presentation by MHCII to CD4 T cells would not be desirable in this location due to high cytokines levels directly entering blood.

### Characterisation of spleen stromal cell lines

Most information on the stromal cell contribution to spleen development and hematopoiesis comes from conditional deletion studies using mutant mice, combined with immunocytochemical identification of changes in cell and tissue composition. These indirect studies are highly informative, but the definition of stromal cell function needs to be supported by studies on isolated cells. The purification of stromal cells through cell dissociation is however fraught with difficulty and is limited by known marker expression. Early studies to isolate mesenchymal stem cells showed that culturing bone marrow stroma was sufficient to capture these rare cells amongst stroma which grew *in vitro* (Muraglia et al., 2000). Such stromal cell studies are rare for bone marrow and almost non-existent for spleen.

As a prelude to *ex vivo* characterisation of splenic stromal cells which support hematopoiesis, stromal cells lines were analysed for characteristics indicating their lineage origin. Cell surface phenotyping of several cloned lines including 5G3 and 3B5 showed expression of the CD105, CD29, CD90 and PDPN (gp38) markers of SSPCs, and the PDGFRA, CD106 and CD51 markers of perivascular reticular cells (O'Neill et al., 2019; Lim et al., 2018). Absence of CD31, CD54 and CD45 expression ruled out an endothelial or hematopoietic lineage origin. Transcriptome analysis of stromal lines confirmed the mesenchymal origin of cells and their resemblance to mesenchymal stem cells through expression of *Col1a1, Sca1, Pdpn, Cd164, Cd90, Cd2*9 and *Cd106* (O'Neill et al., 2019). High expression of genes like *Mmp3, Cxcl12, Pdgfrb, Pdgfra, Nkx2-5, Itgav* and *Scf* reflected perivascular reticular cells described in bone marrow (Ding et al., 2012), although absence of *Nes, Mcam, Lepr* and *Nte5* expression distinguished them from their bone marrow counterparts (Ding et al., 2012; Asada et al., 2017b).

Production of CXCL12 and SCF by 5G3 stromal cells is consistent with their capacity to support hematopoiesis (Lim et al., 2018). Their important role in hematopoiesis was confirmed through addition of inhibitors of HSC signalling to co-cultures. Inhibitors for Notch and Wnt signalling pathways or inhibitors of SCF and CXCL12 receptor uptake, block *in vitro* cell differentiation of HSCs over 5G3 stroma (Lim et al., 2018). Expression of adhesion molecules like VCAM1 by stroma is also consistent with their interaction with HSCs expressing VLA-4, a signalling pathway which supports HSC maintenance and differentiation (Ulyanova et al., 2005; Martinez-Agosto et al., 2007; Castagnaro et al., 2013). Stroma also express SPP1 consistent with their role in hematopoiesis since SPP1 binding to CD44 on HSC maintains their quiescent state (Nilsson et al., 2005; Stier et al., 2005). In sum, our work shows that spleen contains stromal cells reflecting perivascular reticular cells and SSPCs which express receptors for signalling HSCs.

### Identification of splenic stromal cells as osteoprogenitors

A collection of recent studies now identifies SSPCs as a heterogeneous population of multi-lineage progenitors with distinct differentiative capacity. The skeletal stem cell isolated from both fetal bone of mice and humans is multipotent with capacity to form osteoblasts, chondrocytes and stromal cells, but not adipocytes (Chan et al., 2013; Chan et al., 2015; Chan et al., 2018). Mesenchymal stem cells have been isolated from mouse bone marrow which also have osteo-chondrogenic differentiative capacity and these cells were also shown to support maintenance of cord blood-derived primitive HSC when stroma was grown *in vitro* (Matsuoka et al., 2015). A multitude of studies on bone marrow stromal cells which support hematopoiesis favour the existence of mesenchymal cells which are adipogenic and retain some osteogenic differentiative potential (Shen et al., 2008). In organs outside of bone and bone marrow such as adipose tissue, microvascular pericytes and adventitial perivascular cells are observed to include multi-lineage progenitors which are active in tissue turnover in response to pathological remodelling. SSPC subsets have been isolated as perivascular cells which exist in the tunica adventitia of arteries and veins (Xu et al., 2021), and several distinct populations were identified through marker expression. Of interest here are the PDGFRA-expressing cells which are distinct as SSPCs in that they have restricted osteogenic capacity (Wang et al., 2020), and thus differ from the SSPC subset dominant in bone which reflects a progenitor with osteogenic, chondrogenic and stroma-forming differentiative capacity (Chan et al., 2015; Chan et al., 2018).

Very few studies have been performed on spleen to identify any inherent SSPC subsets. However, the isolated spleen stromal lines 5G3 and 3B5 were found to resemble skeletal progenitors since they have osteogenic differentiative capacity when cultured under mineralisation conditions, but lacked capacity for chondrogenesis or adipogenesis (O'Neill et al., 2019). These stromal lines were also shown to express genes reflecting early osteogenic precursors like *Spp1, Col1a2, Mmp2, Bmp2, Cdh11* and *Fn*, although not genes of mature osteoblasts including *Sp7, Cbfa1, Alpl, Bglap2* and *Ibsp* (O'Neill et al., 2019). This evidence raises the hypothesis that spleen contains a unique perisinusoidal niche comprising stromal cells resembling osteoprogenitors that support extramedullary hematopoiesis. The unexpected finding of an osteoblastic progenitor cell in spleen suggests a specific subset of SSPCs with an important function in supporting HSCs and their differentiation in proximity to sinusoids in the red pulp.

Using information on the phenotype of splenic stromal cell lines which support hematopoiesis, we undertook a large project to identify and isolate multiple stromal fractions *ex vivo*, assess their phenotype, and to test their growth and hematopoietic support capacity. These same subsets were also tested for capacity to form a spleen stromal graft when transplanted under the kidney capsule. Since long-term stromal cultures were best established with neonatal 6-day spleens, neonatal tissues were used for cell isolation. Spleens were fractionated to remove red blood cells and hematopoietic cells, and then sorted on the basis of expression of markers for mesenchymal stem cells (CD29, PDPN, CD105, PDGFRA, CD90), endothelial cells (CD31, VCAM1), perivascular reticular cells (CD146, MAdCAM1) and mature spleen stromal cells (SCA1, CD51, ER-TR7) (Lim and O'Neill, 2019). On the basis of capacity to form a confluent monolayer of stromal cells by 28 days, only subsets expressing the CD29, PDPN, CD105, PDGFRA and CD90 markers of mesenchymal stem cells, or lacking the endothelial markers CD31 and VCAM1, formed confluent monolayers (Lim and O'Neill, 2019). Other fractionations based on mature stromal markers (SCA1, CD51, ER-TR7) or perivascular reticular cell markers (CD146, MAdCAM1) were less informative. Each of the cell lines which grew acquired the same phenotype after 28 days of culture, reflecting mesenchymal stem/progenitor cells as SCA1^+^ PDPN^+^ CD51^+^ CD105^+^ PDGFRA^+^ CD90^+^ ER-TR7^-^ (Lim and O'Neill, 2019). Indeed, this outgrowth of a common mesenchymal cell type was demonstrated previously in culture of bone marrow stroma, with outgrowth of a common similar mesenchymal stem cell type (Muraglia et al., 2000).

Cell lines derived *in vitro* from 28-day cultures of spleen stromal fractions were also shown to support hematopoiesis when overlaid with hematopoietic stem/progenitor cells (Lim and O'Neill, 2019). Most established stroma supported the production of myeloid cells equivalent to those produced in control 5G3 stromal cultures, although cell production levels were lower and more variable. Several stromal subsets, including the SCA1^lo^CD90^lo^CD105^+^CD51^+^CD140A^+^ and SCA1^lo^CD90^−^CD105^+^CD51^lo^CD140A^lo^ cells, were identified to grow well and to produce monolayers which were strong supporters of myelopoiesis (Lim and O'Neill, 2019).

These same fractionated stromal subsets were also tested *in vivo* for capacity to form stromal grafts when transplanted under the kidney capsule. Previously it had been shown that spleen capsular tissue could engraft to form a spleen graft which became filled with hematopoietic cells from the host (Tan and Watanabe, 2014). Extensive experiments were performed using dissociated capsular tissue and fractionation of specific subsets based on marker expression. However, engraftment was found to be universally unsuccessful using dissociated and fractionated splenic stromal cells. Subsequent experiments involving engraftment of several long-term stromal lines into NOD/SCID host kidney were found to be successful (O'Neill et al., 2019). These stromal lines formed ectopic niches for hematopoiesis, evident specifically by the presence of myeloid cells similar to those produced in *in vitro* co-cultures of hematopoietic progenitors above stroma, and because HSC could be detected within ectopic grafts (Adolfsson et al., 2005).

## Conclusion and outlook

This report describes an SSPC subset in spleen which presents as perivascular reticular cells which support extramedullary hematopoiesis and specifically myelopoiesis. Extramedullary hematopoiesis is an important alternative pathway for hematopoiesis which occurs following stress, infection and bone marrow compromise. This perivascular stromal cell type plays an important role in remodelling of spleen and HSC niches after stress and with ageing and disease. It remains an important cell target for regenerative medicine to replace or amplify damaged niches. Indeed, ageing of SSPCs and the niche they provide for HSCs in bone marrow has been identified as a cause for decline or skewing of blood and bone lineages (Ambrosi et al., 2021).

Mesenchymal stromal cells which form HSC niches in spleen are distinct from those which form niches in bone marrow, raising questions around the equivalence of HSC subsets maintained in those organs and their hematopoietic contribution. Splenic stromal cells which form HSC niches reflect SSPC with osteogenic capacity and are associated with sinusoids in the red pulp region. They differ from the most common stromal subset of perisinusoidal stroma which supports HSC quiescence in bone marrow and which has adipogenic differentiative capacity. Splenic perisinusoidal reticular cells do not express the LepR and Nestin markers of stroma which form the HSC niche in bone marrow, and are distinct through expression of Tlx1 and Tcf21. Stromal niche elements in bone marrow and spleen express markers of mesenchymal progenitors and produce high levels of SCF and CXCL12.

As a secondary lymphoid organ, the spleen has remarkable capacity to undergo continuous remodelling of the stromal microenvironment to facilitate immune responses (Golub et al., 2018). Spleen also has remarkable regenerative capacity (Tan and Watanabe, 2014; Tan and Watanabe, 2017), such that spleen tissue fragments can be successfully grafted under murine kidney capsule for development of ectopic spleen tissue showing both red and white pulp formation and full hematopoietic reconstitution (Tan and Watanabe, 2014). Engraftment of stromal fractions isolated by enrichment based on cell surface markers has also led to identification of two cell types necessary for spleen regeneration. A spleen organiser cell was identified as an endothelial-like CD31^+^MAdCAM-1^+^ cell, and a second cell type was found to be a mesenchymal PDGFRβ^+^ cell, consistent with the requirement of a mesenchymal stromal cell in formation of niches for HSCs (Tan and Watanabe, 2014; Tan and Watanabe, 2017; Deng et al., 2018). Indeed, the remarkable regenerative capacity of spleen could contribute to recovery of HSC niches following myeloablative damage. The effect of myeloablation or irradiation on splenic niches for HSCs is not well documented, despite common use of these procedures.

## Data Availability

The original contributions presented in the study are included in the article, further inquiries can be directed to the corresponding author.
